# PLK1 and its substrate MISP facilitate intrahepatic cholangiocarcinoma progression by promoting lymphatic invasion and impairing E-cadherin adherens junctions

**DOI:** 10.1038/s41417-023-00705-z

**Published:** 2023-12-06

**Authors:** Yi-Ru Pan, Joseph Chieh-Yu Lai, Wen-Kuan Huang, Pei-Hua Peng, Shih-Ming Jung, Sheng-Hsuan Lin, Chiao-Ping Chen, Chiao-En Wu, Tsai-Hsien Hung, Alice L. Yu, Kou-Juey Wu, Chun-Nan Yeh

**Affiliations:** 1 Department of Surgery, Chang Gung Memorial Hospital at Linkou, Chang Gung University, Taoyuan, 333 Taiwan; 2 Cancer Genome Research Center, Chang Gung Memorial Hospital at Linkou, Taoyuan, 333 Taiwan; 3 Institute of Biomedical Sciences, China Medical University, Taichung, 404 Taiwan; 4 Division of Hematology-Oncology, Department of Internal Medicine, Chang Gung Memorial Hospital at Linkou, Chang Gung University College of Medicine, Taoyuan, 333 Taiwan; 5 Department of Pathology, Chang Gung Memorial Hospital at Linkou, Taoyuan, 333 Taiwan; 6 Institute of Stem Cell and Translational Cancer Research, Chang Gung Memorial Hospital at Linkou, Chang Gung University, Taoyuan, 333 Taiwan; 7 Department of Pediatrics, University of California in San Diego, San Diego, CA 92103 USA; 8 School of Medicine, National Tsing Hua University, Hsinchu, 30013 Taiwan

**Keywords:** Cancer, Targeted therapies

## Abstract

Intrahepatic cholangiocarcinoma (iCCA) is a subtype of CCA and has a high mortality rate and a relatively poor prognosis. However, studies focusing on increased cell motility and loss of epithelial integrity during iCCA progression remain relatively scarce. We collected seven fresh tumor samples from four patients to perform RNA sequencing (RNA-seq) and assay for transposase-accessible chromatin using sequencing (ATAC-seq) to determine the transcriptome profile and chromatin accessibility of iCCA. The increased expression of cell cycle regulators, including PLK1 and its substrate MISP, was identified. Ninety-one iCCA patients were used to validate the clinical significance of PLK1 and MISP. The upregulation of PLK1 and MISP was determined in iCCA tissues. Increased expression of PLK1 and MISP was significantly correlated with tumor number, N stage, and lymphatic invasion in an iCCA cohort. Knockdown of PLK1 or MISP reduced trans-lymphatic endothelial migration and wound healing and affected focal adhesions in vitro. In cell‒cell junctions, MISP localized to adherens junctions and suppressed E-cadherin dimerization. PLK1 disrupted adherens junctions in a myosin-dependent manner. Furthermore, PLK1 and MISP promoted cell proliferation in vitro and tumorigenesis in vivo. In iCCA, PLK1 and MISP promote aggressiveness by increasing lymphatic invasion, tumor growth, and motility through the repression of E-cadherin adherens junctions.

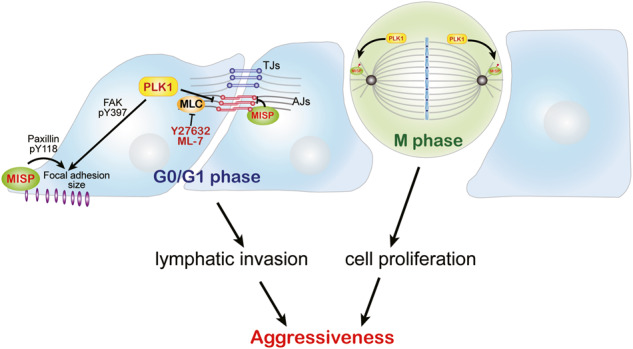

## Introduction

Cholangiocarcinoma (CCA) is a malignant tumor from cholangiocytes in bile duct branches and is the second most common aggressive malignancy in the liver. There are three types: intrahepatic cholangiocarcinoma (iCCA), perihilar cholangiocarcinoma (pCCA), and distal cholangiocarcinoma (dCCA) [1, 2]. CCA has a high mortality rate and a relatively poor prognosis [3, 4]. Several risk factors have been demonstrated to affect overall survival (OS), including tumor size, tumor number, vascular infiltration, differentiation, vascular invasion, lymphatic invasion, and perineural invasion [5–8]. The major lymphatic invasion/metastatic processes are the detachment of cancer cells from the primary tumor, their penetration of lymphatic endothelial cells, and the survival and growth of these tumor cells in the lymph nodes [9, 10]. Thus, cell plasticity, proliferation, and survival are associated with the degree of lymphatic invasion.

Recently, high-throughput genome-wide next-generation sequencing (NGS) technologies have been widely used to profile gene mutations and expression occurring in cancer. To characterize the genomic landscape of CCA and provide insights into its biology, an increasing number of studies have applied NGS approaches to CCA [11, 12]. RNA sequencing combined with ATAC sequencing (Assay for Transposase Accessible Chromatin with sequencing) could be utilized in the profiling of chromatin accessibility regions in different types of human cancers that can allow the discovery of biomarkers and predict patient prognosis.

Polo-like kinases (PLKs) are serine/threonine protein kinases [13] that contain five family members, namely, *PLK1, PLK2, PLK3, PLK4*, and *PLK5* [14]. Among these members, PLK1 plays a crucial role in the cell cycle, cell division, and DNA damage response [15–17]. Recent studies have shown that PLK1 contributes to carcinogenesis by mediating apoptosis [17], autophagy [18], the immune response [19], inflammation [20], and epithelial-to-mesenchymal transition (EMT) [21]. In EMT, PLK1 regulates kinases or transcription factors (MAPK, Akt, c-Raf, and FoxM1) to activate EMT transcription factors that trigger the EMT process [21, 22]. In many types of cancer, the expression of PLK1 is upregulated, and its overexpression is usually associated with poor patient survival [23], including melanoma [24], breast cancer [25], and prostate cancer [26]. In CCA, the PLK1 inhibitor BI-2536 repressed CCA tumor growth [27].

MISP (Mitotic Spindle Positioning, C19orf21, Caprice) is an actin-associated protein. In mitosis, PLK1 directly phosphorylated MISP to mediate spindle orientation by linking microtubules to actin filaments [28–30] and activating the complex of IQGAP1 and Cdc42 [31]. In epithelial microvilli, MISP acted as an actin-bundling protein to stabilize microvillar rootlets and recruited fimbrin to promote the elongation of microvillar rootlets [32]. In pancreatic ductal adenocarcinoma, MISP promoted cell proliferation, migration, and invasion in vitro [33]. However, the role of MISP in tumor lymphatic invasion and EMT has not been demonstrated.

Regarding cell‒cell junctions, there are four types: tight junctions (TJs), adherens junctions (AJs), desmosomes, and gap junctions [34]. TJs and AJs are modulated by actin architectures [35]. TJs mainly mediate the passage of small molecules and ions, and AJs regulate cell‒cell adhesion [36]. Lateral interactions through the extracellular domain of cadherin are essential for the integrity of AJs [37]. In a previous report, the networks of myosin activation, actin dynamics, central spindle organization, and cytokinesis were found to involve the PLK1 Polo-box domain (PDB) interactome [38]. PLK1 promotes contraction by indirectly regulating myosin light chain (MLC) phosphorylation during mitosis [39]. Actomyosin contractility regulates AJs [40], and unbalanced contractile stresses affect the integrity of AJs [41]. The phosphorylation of MLC is modulated by ROCK (Rho-associated protein kinase) and MLC kinase [42]. The role of PLK1 and MISP in regulating tumor cell adhesive functions remains to be determined.

In this study, we identified the upregulation of PLK1 and MISP in CCA patient tumor tissues and further uncovered the mechanism by which PLK1 and MISP mediate lymphatic invasion in iCCA.

## Materials and methods

### Patient samples

**Fig. 1:**
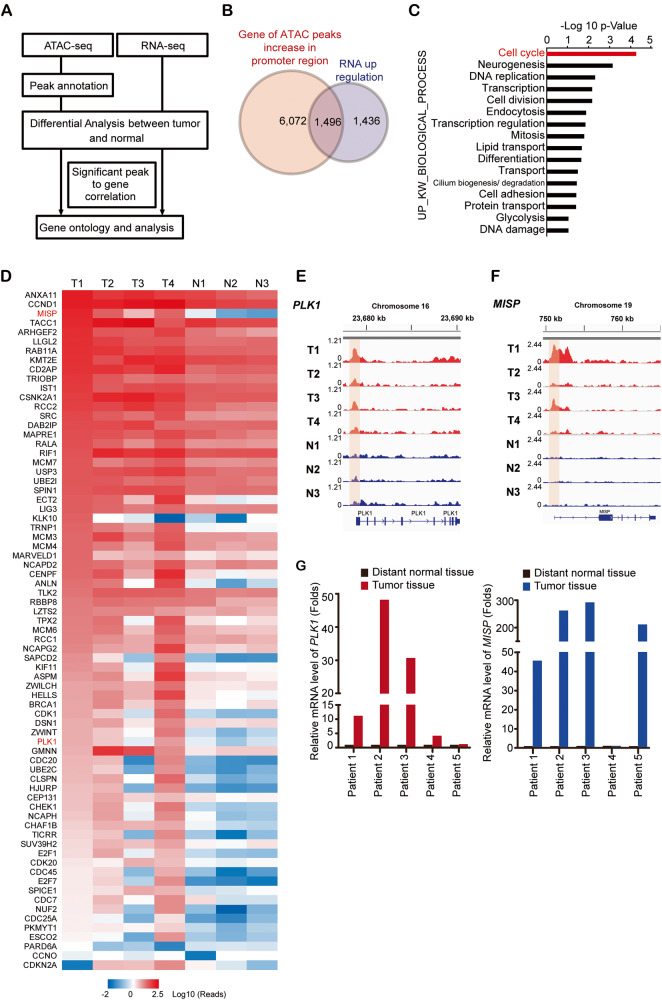
The expression of PLK1 and its substrate MISP was upregulated in intrahepatic cholangiocarcinoma (iCCA). **A** A schema showing the process for identifying the 1496 genes for the analysis. **B** A total of 1496 genes were identified from the intersection of 7568 increased accessibility regions by ATAC-seq analysis and 2932 upregulated genes by RNA-seq analysis. **C** The biological process analysis of the increased genes in Supplementary Table 2 shows the possible pathways involved in iCCA carcinogenesis. **D** A heatmap analysis of gene expression showed a ranking of cell cycle gene expression in tumor samples (T1, T2, T3, and T4) and their distant normal liver samples (N1, N2, and N3) from iCCA patients. **E** Comparison of representative ATAC-seq gene tracks at the *PLK1* locus from 3 normal tissues and 4 tumor tissues from iCCA patients. **F** Comparison of representative ATAC-seq gene tracks at the *MISP* locus from 3 normal tissues and 4 tumor tissues from iCCA patients. **G** The relative mRNA levels of *PLK1* and *MISP* in tumor samples (red: *PLK1*; blue: *MISP*) and their distant normal liver samples (black) from five iCCA patients. The values are presented as the fold change relative to the level of their distant normal liver samples.

**Fig. 2:**
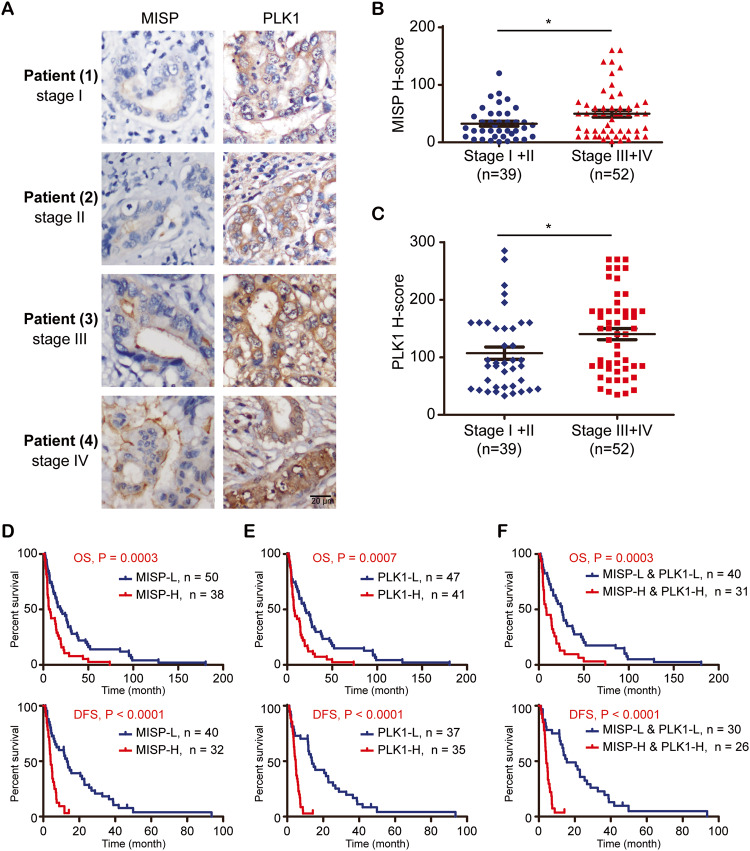
The expression levels of PLK1 and MISP were associated with poor prognosis and were increased in patients with late*-*stage iCCA. **A** Representative pictures of immunohistochemical staining of MISP and PLK1 in iCCA specimens. The clinical stage of each patient is indicated. **B** Distribution of the H score for MISP expression from iCCA patients (*n* = 91). *P < 0.05 by Student’s *t* test. **C** Distribution of the H score for PLK1 expression from iCCA patients (*n* = 91). *P < 0.05 by Student’s *t* test. **D** Kaplan‒Meier plots of overall survival (OS) and disease-free survival (DFS) of iCCA patients with high MISP expression (MISP-H; >mean) and low MISP expression (MISP-L; <mean). The *P* values shown in the panel were determined by the log*-*rank test. **E** Kaplan‒Meier plots of OS and DFS of iCCA patients with high PLK1 expression (PLK1-H; >mean) and low PLK1 expression (PLK1-L; <mean). The *P* values shown in the panel were determined by the log*-*rank test. **F**. Kaplan‒Meier plots of OS and DFS of iCCA cancer patients with both high PLK1 and high MISP expression and both low PLK1 and low MISP expression. The *P* values shown in the panel were determined by the log*-*rank test.

Seven fresh tissues from four patients for RNA sequencing (RNA-seq) and assay for transposase-accessible chromatin using sequencing (ATAC-seq) and ten fresh tissues from five patients for RT‒qPCR were pathologically confirmed as iCCA samples retrieved from Linkou Chang Gung Memorial Hospital (Fig. 1). Ninety-one paraffin-embedded specimens presented in Fig. 2, Supplementary Fig. 2, and Table 1 were pathologically confirmed iCCA samples retrieved from Linkou Chang Gung Memorial Hospital. The study was approved by the Institutional Review Board Linkou Chang Gung Memorial Hospital (IRB 201900137B0). Immunohistochemistry was performed as previously described [43]. In brief, a 4‑μm section was stained for PLK1 and MISP. Primary antibodies (Supplementary Fig. 4) were incubated overnight at 4 °C and visualized using the REAL EnVision Detection System, Peroxidase/DAB+, Rb/Mo (K500711, DAKO, Agilent Technologies, Inc., Santa Clara, California, United States). H scores were analyzed under microscopy by the authors and calculated by multiplying the intensity by the percentage of the positive area. Sample sizes were determined by data availability.

**Table 1:** Association of PLK1 and MISP expression with clinical-pathological parameters in 91 iCCA patients.

Characteristics	*n* (%)	MISP1 H-score			PLK1 H-score		
		Low	High	*P*	Low	High	*P*
		(*n* = 52)	(*n* = 39)	value	(*n* = 49)	(*n* = 42)	value
Gender				0.853			0.869
Male	36 (39.6)	21 (40.4)	15 (38.5)		19 (38.8)	17 (40.5)	
Female	55 (60.4)	31 (59.6)	24 (61.5)		30 (61.2)	25 (59.5)	
Age (years)				0.447			0.223
≦65	59 (64.8)	32 (61.5)	27 (69.2)		29 (59.2)	30 (71.4)	
>65	32 (35.2)	20 (38.5)	12 (30.8)		20 (40.8)	12 (28.6)	
HBV infection				0.777			0.29
No	69 (75.8)	40 (76.9)	29 (74.4)		35 (71.4)	34 (81.0)	
Yes	22 (24.2)	12 (23.1)	10 (25.6)		14 (28.6)	8 (19.0)	
HCV infection				0.507			**0.018**
No	81 (89.0)	45 (86.5)	36 (92.3)		40 (81.6)	41 (97.6)	
Yes	10 (11.0)	7 (13.5)	3 (7.7)		9 (18.4)	1 (2.4)	
Total bilirubin (mg/dL)				0.807			0.542
≦1.2	76 (83.5)	43 (82.7)	33 (84.6)		42 (85.7)	34 (81.0)	
>1.2	15 (16.5)	9 (17.3)	6 (15.4)		7 (14.3)	8 (19.0)	
Albumin (g/dL)				0.131			0.514
≦3.5	21 (23.1)	9 (17.3)	12 (30.8)		10 (20.4)	11 (26.2)	
>3.5	70 (76.9)	43 (82.7)	27 (69.2)		39 (79.6)	31 (73.8)	
CEA (ng/mL)^a^				0.944			0.507
≦5	38 (43.7)	22 (44.0)	16 (43.2)		19 (40.4)	19 (47.5)	
>5	49 (56.3)	28 (56.0)	21 (56.8)		28 (59.6)	21 (52.5)	
Margin				0.066			**0.021**
Negative	63 (69.2)	40 (76.9)	23 (59.0)		39 (79.6)	24 (57.1)	
Positive	28 (30.8)	12 (23.1)	16 (41.0)		10 (20.4)	18 (42.9)	
Tumor no.				**<0.001**			**0.013**
Single	73 (80.2)	49 (94.2)	24 (61.5)		44 (89.8)	29 (69.0)	
Multiple	18 (19.8)	3 (5.8)	15 (38.5)		5 (10.2)	13 (31.0)	
Gross type				0.07			0.457
MF	61 (67.0)	39 (75.0)	22 (56.4)		34 (69.4)	27 (64.3)	
MF + PI	19 (20.9)	10 (19.2)	9 (23.1)		8 (16.3)	11 (26.2)	
PI	11 (12.1)	3 (5.8)	8 (20.5)		7 (14.3)	4 (9.5)	
Tumor size (cm)				0.096			0.188
≦5	37 (40.7)	25 (48.1)	12 (30.8)		23 (46.9)	14 (33.3)	
>5	54 (59.3)	27 (51.9)	27 (69.2)		26 (53.1)	28 (66.7)	
T stage				0.102			0.13
T1	39 (42.9)	27 (51.9)	12 (30.8)		25 (51.0)	14 (33.3)	
T2	17 (18.6	7 (13.5)	10 (25.6)		6 (12.2)	11 (26.2)	
T3/4	35 (38.5)	18 (34.6)	17 (43.6)		18 (36.7)	17 (40.5)	
N stage				**0.001**			**0.012**
N0	60 (65.9)	42 (80.8)	18 (46.2)		38 (77.6)	22 (52.4)	
N1	31 (34.1)	10 (19.2)	21 (53.8)		11 (22.4)	20 (47.6)	
Histology				0.077			0.059
Differentiated	51 (56.0)	25 (48.1)	26 (66.7)		23 (46.9)	28 (66.7)	
Undifferentiated	40 (44.0)	27 (51.9)	13 (33.3)		26 (53.1)	14 (33.3)	
Vascular invasion				0.525			0.372
No	75 (82.4)	44 (84.6)	31 (79.5)		42 (85.7)	33 (78.6)	
Yes	16 (17.6)	8 (15.4)	8 (20.5)		7 (14.3)	9 (21.4)	
Lymphatic invasion				**0.011**			0.248
No	72 (79.1)	46 (88.5)	26 (66.7)		41 (83.7)	31 (73.8)	
Yes	19 (20.9)	6 (11.5)	13 (33.3)		8 (16.3)	11 (26.2)	
Perineural invasion				**0.019**			0.155
No	59 (64.8)	39 (75.0)	20 (51.3)		35 (71.4)	24 (57.1)	
Yes	32 (35.2)	13 (25.0)	19 (48.7)		14 (28.6)	18 (42.9)	
Recurrence				0.384			0.1
No	10 (11.0)	7 (13.5)	3 (7.7)		8 (16.3)	2 (4.8)	
Yes	81 (89.0)	45 (86.5)	36 (92.3)		41 (83.7)	40 (95.2)	

Bold values indicates statistical significant *P* values (*P* < 0.05).

^a^Data not available in 4 patients.

*CEA* carcinoembryonic antigen, *HBV* hepatitis B virus, *HCV* hepatitis C virus.

### Cell lines

RBE cells were purchased from the RIKEN Cell Bank (Ibaraki, Japan). MMNK-1, KKU-213, and HuCCT1 cells were purchased from the Japanese Collection of Research Bioresources Cell Bank (Tokyo, Japan). Human dermal lymphatic endothelial cells (HDLECs) were purchased from PromoCell (C-12217, Heidelberg, Germany). Mouse CCA AKP-M1 cells were previously described [44]. RBE cells, HuCCT1 cells, and HuCCT1-derived sublines (N1, N2, N3, and N4) were grown in RPMI medium supplemented with 10% fetal bovine serum (FBS) and penicillin‒streptomycin. The MMNK1, KKU-213, and AKP-M1 cell lines were grown in DMEM supplemented with 10% FBS and penicillin‒streptomycin. HDLECs were grown in endothelial cell growth medium MV 2 (C-22022, PromoCell). All the cell lines used in this study were tested for mycoplasma contamination and authenticated by the short tandem repeat (STR) method.

### Animal experiments

All procedures were conducted according to the institutional animal welfare guidelines of Chang Gung Memorial Hospital. The sample size was estimated from previous studies in tumor-bearing mice [44]. BALB/c nude mice were purchased from BioLASCO Taiwan Co., Ltd. HuCCT1 cells (5 × 10^6^) were injected into the subcutaneous tissue of BALB*/*c nude mice. When the tumor volume reached 100–150 mm^3^, the mice were divided into four groups according to the tumor sizes of the baseline. The mice were then injected with 10 mg/kg volasertib (twice a week, intravenous injection), 10 mg/kg gemcitabine (once a week, intraperitoneal injection), or 0.9% NaCl (twice a week, intravenous injection) for another three weeks. C57BL/6 J mice were purchased from National Applied Research Laboratories Taiwan. AKP-M1 cells (1 × 10^6^) were injected into the right lobes of the livers of C57BL/6 J mice. The mice were divided into four groups according to the IVIS signal intensity of the baseline (Day 5). Volasertib (25 mg/kg) was injected one week after tumor injection. Tumor growth was evaluated by IVIS twice a week for 22 days. The animal experiments were approved by the Institutional Animal Care and Utilization Committee of Chang Gung Memorial Hospital (IACUC 2021082703; IACUC 2023061402).

### Statistics

The results are presented as the means ± SD or SEM. A two-tailed independent Student’s *t* test was used to compare the continuous variables between the two groups. Disease-free survival (DFS) and OS rates were evaluated using the Kaplan–Meier method. Several clinicopathological variables were considered for the initial univariate analysis and multivariate analysis, which were performed using the log-rank test. The Cox proportional hazards model was applied for multivariate regression analysis. The statistical software package SPSS for Windows (SPSS version 17.0, Chicago, IL, USA) was used for statistical analysis. Differences were considered statistically significant at *P* < 0.05 for all of the tests.

## Results

### Increased expression of PLK1 and its substrate MISP in iCCA

To investigate the transcriptome profiling and chromatin accessibility of iCCA, we performed RNA-seq and ATAC-seq on seven samples (Fig. 1A) collected from four iCCA patients (Supplementary Table 1). To compare the variances between samples, we performed principal component analysis (PCA) on RNA-seq datasets and ATAC-seq. The results showed that the tumor samples were grouped compared to the normal liver tissue group (Supplementary Fig. 1A, B). Three normal tissues and four tumor tissues from iCCA patients were analyzed by ATAC-seq and RNA-seq. Comparing the chromatin accessibility between these tumors vs. normal tissues, we identified 7568 regions with significantly increased accessibility (cutoffs were a 1.5-fold change and *p* value < 0.05) in tumor vs. normal tissues (Fig. 1B). Among the 7568 regions, we only focused on 1496 regions that also exhibited upregulated genes in tumor vs. normal tissues (cutoffs were a 1.5-fold change and *p* value < 0.05) in RNA-seq analysis (Fig. 1B). There was a positive correlation between the four iCCA patients and three normal tissues. (Supplementary Fig. 1C). The cell cycle was found at the top of the various pathways for the biological process category of these upregulated genes (Fig. 1C). Heatmap analysis of cell cycle gene expression showed increased expression of PLK1 (polo-like kinase 1) and its substrate MISP (mitotic spindle positioning) in iCCA tumor samples (Fig. 1D). The ATAC-seq tracks from the genome datasets showed high chromatin-accessible regions in the promoters of *PLK1* and *MISP* in tumor tissues (Fig. 1E, F), consistent with the ATAC-seq analysis. We further confirmed the mRNA expression of *PLK1* and *MISP* in five iCCA tumor samples and their adjacent normal liver tissues (Supplementary Table 1). The mRNA levels of *PLK1* and *MISP* were increased in four patients (Fig. 1G). The protein expression of MISP was increased in the iCCA cell lines (RBE, KKU-213 and HuCCT1) compared to the immortalized human cholangiocyte cell line (MMNK1; Supplementary Fig. 1D).

### Clinical impact of the increased expression of PLK1 and MISP in predicting the survival of iCCA patients

Next, we evaluated the impact of PLK1 and MISP expression on iCCA progression using patient specimens (Fig. 2A). The expression levels of PLK1 and MISP were increased in iCCA patients with late-stage disease (stage III and stage IV) compared to those with early-stage disease (stage I and stage II; Fig. 2B, C). iCCA patients who underwent hepatectomy with high expression of MISP and PLK1 had inferior OS and DFS compared with those with low expression (Fig. 2D–F and Supplementary Fig. 2). Regarding clinicpathological parameters, the increased expression of MISP or PLK1 was significantly correlated with tumor number, N stage, and lymphatic invasion (Table 1), indicating that its impact on iCCA progression and high expression of PLK1 and MISP could be used to predict the survival of iCCA patients.

### MISP and PLK1 mediate translymphatic endothelial migration in iCCA cells

**Fig. 3:**
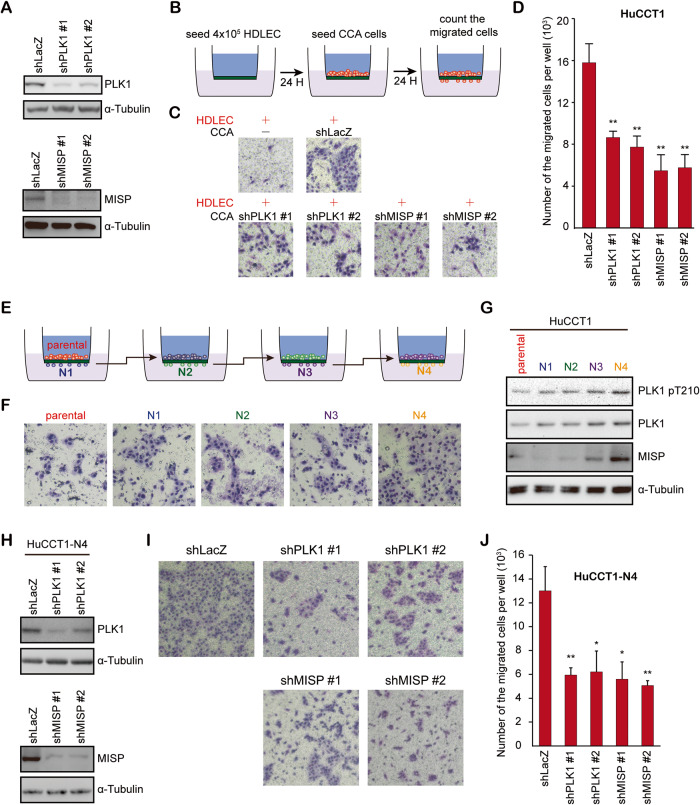
MISP and PLK1-mediated lymphatic invasion in iCCA cells. **A** Western blots showing the levels of PLK1 and MISP in HuCCT1 cells that received shRNAs specific to PLK1 (shPLK1 #1 and #2), MISP (shMISP #1 and #2) or LacZ (shLacZ). α-Tubulin was the loading control. **B** Schema of the experimental procedure for the trans-lymphatic endothelial migration assay. **C** Representative images from the trans-lymphatic endothelial migration of 5 × 10^4^ HuCCT1 cells that received shRNAs specific to PLK1 (shPLK1 #1 and #2), MISP (shMISP #1 and #2) or LacZ (shLacZ). **D** The number of migrated cells was calculated per well. The values (mean ± SD) presented are from three independent experiments. **P < 0.005 by Student’s *t* tests. **E** Schema of the experimental procedure for the selection procedures for the generation of invasive sublines HuCCT1-N1 (N1), HuCCT1-N2 (N2), HuCCT1-N3 (N3), and HuCCT1-N4 (N4) from HuCCT1 (parental). **F** Representative images of the trans-lymphatic endothelial migration of 2 × 10^4^ HuCCT1-derived sublines. **G** Western blots showing the levels of phosphorylated PLK1, total PLK1, and total MISP in HuCCT1-derived sublines. α-Tubulin was the loading control. **H** Western blots showing the levels of PLK1 and MISP in the HuCCT1-N4 subline receiving shRNAs specific to PLK1 (shPLK1 #1 and #2), MISP (shMISP #1 and #2) or LacZ (shLacZ). α-Tubulin was the loading control. **I** Representative images from the translymphatic endothelial migration of 2 × 10^4^ HuCCT1-N4 cells receiving shRNAs specific to PLK1 (shPLK1 #1 and #2), MISP (shMISP #1 and #2) or LacZ (shLacZ). **J** The number of migrated cells was calculated per well. The values (mean ± SD) presented are from three independent experiments. *P < 0.005; **P < 0.005 by Student’s *t* tests.

To confirm the clinicopathological correlation between increased PLK1/MISP expression and N stage/lymphatic invasion, we investigated whether PLK1 or MISP regulated lymphatic invasion using a translymphatic endothelial migration assay [45]. Knockdown of PLK1 or MISP by shRNAs in iCCA cells decreased the number of iCCA cells that migrated through a human lymphatic monolayer (Fig. 3A–D). Furthermore, we generated four sublines (HuCCT1-N1, HuCCT1-N2, HuCCT1-N3, and HuCCT1-N4) from four rounds of selection procedures in vitro (Fig. 3E). The lymphatic invasion ability of parental cells (HuCCT1) and the invasive sublines (HuCCT1-N1, HuCCT1-N2, HuCCT1-N3, and HuCCT1-N4) was confirmed by a translymphatic endothelial migration assay (Fig. 3F). PLK1 phosphorylation, PLK1 expression and MISP expression were upregulated in the HuCCT1-N3 and HuCCT1-N4 sublines (Fig. 3G). To further confirm the effect of PLK1 and MISP on migratory function, PLK1 or MISP was depleted in the HuCCT1-N4 subline (Fig. 3H). The knockdown of PLK1 or MISP reduced the translymphatic endothelial migration ability (Fig. 3I, J), suggesting that PLK1 or MISP expression mediated the lymphatic invasion ability of HuCCT1 cells.

### MISP and PLK1 regulate cell migration in iCCA cells

**Fig. 4:**
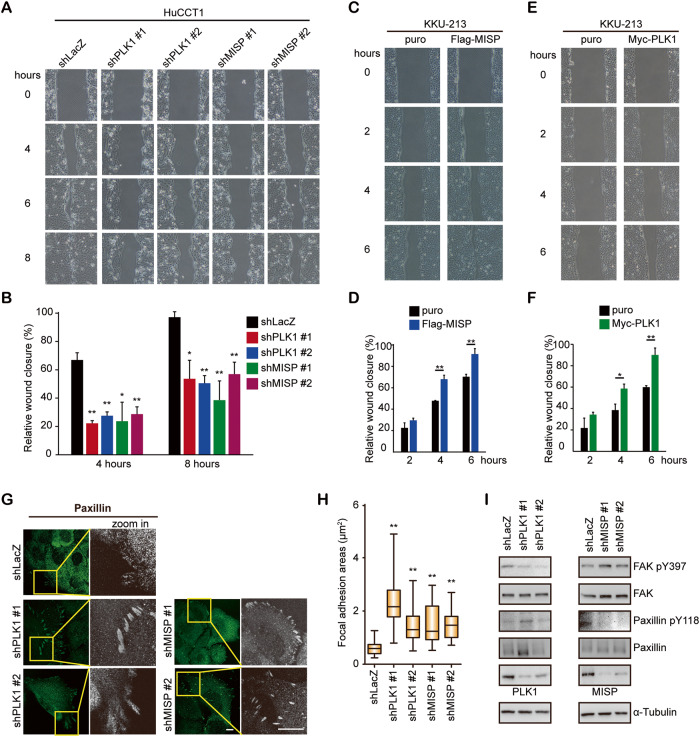
MISP and PLK1 regulated cell migration in iCCA cells. **A** Representative images from the wound-healing assay of HuCCT1 cells that received shRNAs specific to PLK1 (shPLK1 #1 and #2), MISP (shMISP #1 and #2) or LacZ (shLacZ). **B**. The relative wound closure was calculated by analyzing the scratched area covered by the cells after four or eight hours using ImageJ software. The values (means ± SDs) are from three independent experiments and are presented as a percentage relative to the baseline (0 h). *P < 0.05, **P < 0.005 by Student’s *t* test. **C** Representative images from the wound-healing assay of vector (puro) or Flag-tagged wild-type MISP (Flag-MISP)-overexpressing KKU-213 cells. **D** The relative wound closure was calculated by analyzing the scratched area covered by the cells after two, four or six hours using ImageJ software. The values (means ± SDs) are from three independent experiments and are presented as a percentage relative to the baseline (0 h). **P < 0.005 by Student’s *t* test. **E** Representative images from the wound-healing assay of vector (puro) or Myc-tagged PLK1 wild-type (Myc-PLK1)-overexpressing KKU-213 cells. **F** The relative wound closure was calculated by analyzing the scratched area covered by the cells after two, four or six hours using ImageJ software. The values (means ± SDs) are from three independent experiments and are presented as a percentage relative to the baseline (0 h). *P < 0.05; **P < 0.005 by Student’s *t* test. **G** HuCCT1 cells that received shRNAs specific to PLK1 (shPLK1 #1 and #2), MISP (shMISP #1 and #2), or LacZ (shLacZ) were fixed and stained with paxillin (focal adhesion marker). The boxed areas of paxillin from the images are enlarged. Scale bar = 10 µm. **H** The values of the focal adhesion area are from three independent experiments (*n* = 30). Box-and-whisker plots show the distribution of the data: maximum, upper quartile, median, lower quartile, and sample minimum. **P < 0.005 by Student’s *t* test. **I** Western blots showing the levels of phosphorylated FAK, total FAK, phosphorylated paxillin, total paxillin, total PLK1, and total MISP in HuCCT1 cells that received shRNAs specific to PLK1 (shPLK1 #1 and #2), MISP (shMISP #1 and #2) or LacZ (shLacZ). α-Tubulin was the loading control.

To determine the effect of PLK1 or MISP on cell migration, a wound-healing assay was performed. Treatment of HuCCT1 cells with the PLK1 inhibitor volasertib also decreased their cell migration ability (Supplementary Fig. 3A, B). PLK1 or MISP knockdown reduced cell migration in parental HuCCT1 cells (Fig. 4A, B) and the HuCCT1-N4 subline (Supplementary Fig. 3C, D). In contrast, PLK1 or MISP overexpression enhanced cell migration in iCCA KKU-213 cells (Fig. 4C–F and Supplementary Fig. 3E), iCCA HuCCT1 cells (Supplementary Fig. 3F–H) and human cholangiocyte MMNK-1 cells (Supplementary Fig. 3I–K). Since MISP is a focal adhesion-associated protein and interacts with focal adhesion kinase (FAK) [29], focal adhesion morphology was examined. PLK1 knockdown increased the focal adhesion sizes (Fig. 4G, H) and decreased the phosphorylation of FAK Tyr397 (Fig. 4I). MISP knockdown increased the focal adhesion sizes (Fig. 4G, H) and decreased the phosphorylation of paxillin Tyr118 (Fig. 4I), leading to decreased cell migration [46]. Likewise, treatment of HuCCT1 cells with volasertib increased the focal adhesion area (Supplementary Fig. 3L), indicating that MISP or PLK1 may promote cell migration by regulating focal adhesions in iCCA cells.

### Knockdown of PLK1 or MISP enhances E-cadherin adherens junctions in HuCCT1 cells

**Fig. 5:**
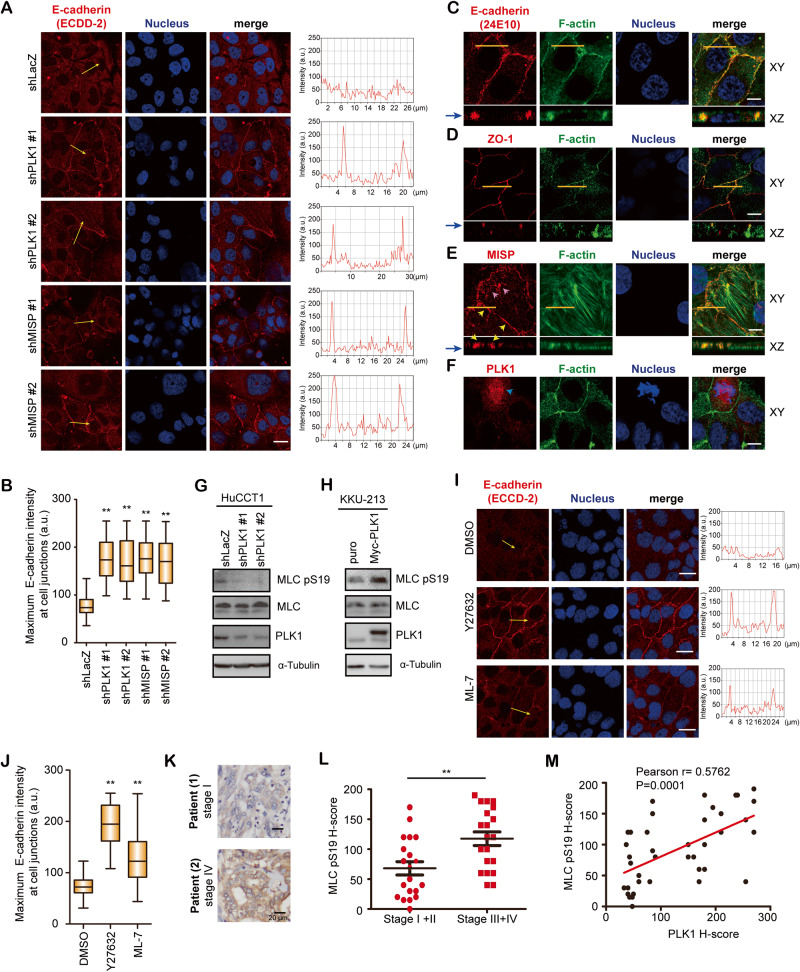
Knockdown of PLK1 or MISP enhanced adherens junctions in HuCCT1 cells. **A** Left: HuCCT1 cells that received shRNAs specific to PLK1 (shPLK1 #1 and #2), MISP (shMISP #1 and #2), or LacZ (shLacZ) were grown to confluence and stained with E-cadherin (ECCD-2, red) and nuclear stain (blue). Scale bar = 20 µm. Right: the profiles show the relative fluorescence intensity of the yellow lines in the E-cadherin images. **B** Quantification of the maximum E-cadherin intensity per cell. The values are from three independent experiments (*n* = 300). Box-and-whisker plots show the distribution of the data: maximum, upper quartile, median, lower quartile, and sample minimum. **P < 0.005 by Student’s *t* tests. **C**–**F** HuCCT1 cells were grown to confluence and stained for E-cadherin (24E10, red, **C**), ZO-1 (red, **D**), MISP (red, **E**), PLK1 (red, **F**), F-actin (green), and nuclear stain (blue). Orange lines on confocal XY section images represent regions where confocal XZ section images were taken. Yellow arrows indicate MISP at cell junctions; pink arrows indicate MISP at focal adhesions; blue arrows indicate PLK1 at spindles. Scale bar = 10 µm. Blue arrows in (**C**), (**D**), and (**E**) indicate the Z positions for the XY images. **G** Western blots showing the levels of the indicated proteins in HuCCT1 cells receiving shRNAs specific to PLK1 (shPLK1 #1 and #2) or LacZ (shLacZ). α-Tubulin was the loading control. **H**. HuCCT1 cells overexpressing vector (puro) or Myc-tagged PLK1 wild type (Myc-PLK1) were arrested at the beginning of S phase using a double thymidine block. Western blots showing the levels of the indicated proteins in the cells. α-Tubulin was the loading control. **I** HuCCT1 cells were grown to confluence and then treated with 10 μM Y27632, 10 μM ML-7 or control DMSO for 24 h. The cells were fixed and stained with E-cadherin (ECCD-2, red) and nuclear stain (blue). Scale bar = 20 µm. The profiles show the relative fluorescence intensity of the yellow lines in the E-cadherin images. **J** Quantification of the maximum E-cadherin intensity per cell. The values are from three independent experiments (*n* = 300). Box-and-whisker plots show the distribution of the data: maximum, upper quartile, median, lower quartile, and sample minimum. **P < 0.005 by Student’s *t* tests. **K** Representative pictures of immunohistochemical staining of MLC phosphorylation (MLC pS19) in iCCA specimens. The clinical stage of each patient is indicated. Scale bar: 20 μm. **L** Distribution of the H score for MLC phosphorylation (MLC pS19) from iCCA patients (*n* = 40). **P < 0.005 by Student’s *t* test. **M** The association between MLC phosphorylation and PLK1 expression in 40 patients. The Pearson correlation coefficient *r* and *P* values are shown in the panel.

To further investigate the possible mechanism of PLK1/MISP-mediated lymphatic invasion, we used an E-cadherin (ECCD-2) antibody to recognize E*-*cadherin dimerization at cell‒cell junctions [47, 48]. Knockdown of PLK1 or MISP increased E*-*cadherin dimerization but did not affect zonula occludens-1 (ZO-1) localization (Fig. 5A, B and Supplementary Fig. 4A). To further clarify how PLK1 and MISP regulated E*-*cadherin dimerization, the localization of PLK1 and MISP was examined. In X-Z vertical sections, we used anti-E-cadherin antibodies (antibody clone 24E10) and anti-ZO-1 antibodies to examine the localization of AJs and TJs, respectively (Fig. 5C, D). MISP was localized at focal adhesions and E-cadherin adherens junctions (Fig. 5D). PLK1 was localized at spindles but not at cell‒cell junctions (Fig. 5F), suggesting that PLK1 may indirectly regulate E*-*cadherin dimerization. PLK1 has been reported to regulate cell motility by inducing EMT [49]. We first detected EMT-related proteins in three iCCA cell lines (RBE, KKU-213, and HuCCT1) and a cholangiocyte cell line (MMNK-1, Supplementary Fig. 4B). However, PLK1 did not consistently regulate these EMT-related proteins when it was knocked down by treatment with two shRNA sequences (Supplementary Fig. 4C). Due to the involvement of myosin light chain kinase and AJs, we hypothesized that PLK1-mediated AJs through myosin. Knockdown of PLK1 reduced the phosphorylation of MLC in HuCCT1 and KKU-213 cells (Fig. 5G and Supplementary Fig. 4D). Overexpression of PLK1 increased MLC phosphorylation (Fig. 5H). Since the phosphorylation of MLC was modulated by ROCK (Rho-associated protein kinase) and MLC kinase [42], a ROCK inhibitor and MLC kinase inhibitor were tested. Treatment of HuCCT1 cells with the ROCK inhibitor Y27632 or the MLC kinase inhibitor ML-7 increased E*-*cadherin dimerization (Fig. 5I, J). In iCCA patients, the phosphorylation of MLC was increased in patients with late-stage disease (stage III and stage IV) compared to that in patients with early-stage disease (stage I and stage II; Fig. 5K, L). MLC phosphorylation positively correlated with the expression of PLK1 (Fig. 5M). These data indicate that MISP may directly localize at AJs to mediate E*-*cadherin dimerization and that PLK1 may regulate AJs by controlling myosin activation.

### Knockdown of PLK1 or MISP impairs tumor growth in vitro and in vivo

**Fig. 6:**
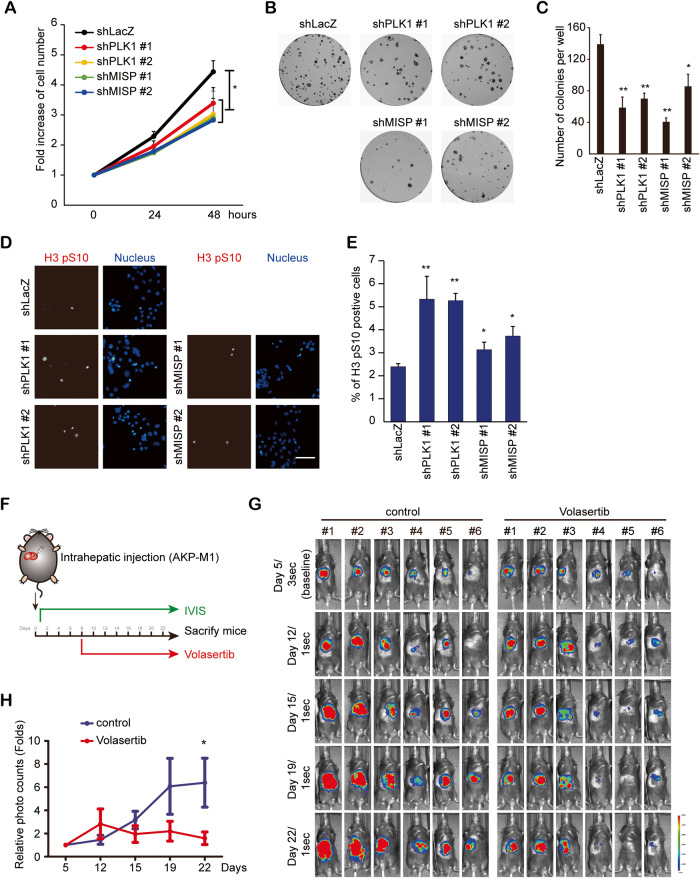
Knockdown of PLK1 or MISP impaired tumor growth in vitro and in vivo. **A** A cell proliferation assay. HuCCT1 cells that received shRNAs specific to PLK1 (shPLK1 #1 and #2), MISP (shMISP #1 and #2) or LacZ (shLacZ) were seeded in a 96-well plate for 0, 24 or 48 h. Cell viability was quantified by the CCK-8 assay. The values (means ± SDs) are from three independent experiments and are presented as the fold change relative to the baseline (0 h). *P < 0.05 by Student’s *t* test. **B** Representative images of the colony formation assay. HuCCT1 cells (500 cells/well) that received shRNAs specific to PLK1 (shPLK1 #1 and #2), MISP (shMISP #1 and #2) or LacZ (shLacZ) were seeded in a six-well plate for 14 days. **C** The values (means ± SDs) are from three independent experiments; *P < 0.05, **P < 0.005 by Student’s *t* test. **D** HuCCT1 cells that received shRNAs specific to PLK1 (shPLK1 #1 and #2), MISP (shMISP #1 and #2) or LacZ (shLacZ) were fixed and stained with Histone 3 Ser10 phosphorylation (H3 pS10) and nuclear stain. Scale bar = 100 µm. **E** Quantification of the p-Ser10 H3-positive cells (*n* > 1000). The values (means ± SDs) are from three independent experiments. *P < 0.05; **P < 0.005 by Student’s *t* test. **F** Schema showing the experimental design of the mouse experiment. **G** AKP-M1 cells were injected into the right lobes of the livers of C57BL/6J mice. Volasertib (25 mg/kg) was injected twice a week after one week of tumor injection. Mouse bioluminescent signals were detected on the indicated days after tumor injection. The exposure times for images on the indicated days are indicated. **H** The relative bioluminescent intensities (means ± SEMs) from six mice are presented as the fold change relative to the baseline (Day 5). *P < 0.05 by Student’s *t* test.

PLK1 is a crucial molecule for cell cycle regulation that promotes tumor proliferation in several cancer types [15]. MISP is directly phosphorylated by PLK1 to control spindle orientation during mitosis [28]. We wanted to confirm the role of PLK1 and MISP in the tumor growth of iCCA cells. Knockdown of PLK1 or MISP decreased the proliferation of HuCCT1 and KKU-213 cells (Fig. 6A and Supplementary Fig. 5A, B) and repressed colony formation (Fig. 6B, C). The phosphorylation of histone H3 at Ser10 was increased during mitosis [50]. The knockdown of PLK1 or MISP increased the percentage of p-Ser10 histone H3-positive cells (Fig. 6D, E), which may result from the depletion of PLK1 or MISP-induced mitotic arrest [29, 51]. In vivo, mouse iCCA AKP-M1 cells [44] were injected into the left lobes of the livers of C57BL/6J mice. Treatment with the PLK1 inhibitor volasertib (25 mg/kg) suppressed tumor growth (Fig. 6F–H). In addition, treatment of xenografted HuCCT1 cells with low-dose volasertib (10 mg/kg) repressed tumor growth, and a combination of low-dose volasertib and low-dose GEM (10 mg/kg) treatment further decreased tumor growth (Fig. 6D), supporting the role of PLK1 and MISP in regulating cell proliferation in vitro and tumorigenesis in vivo in iCCA cells.

## Discussion

In this study, increased expression of PLK1 and its substrate MISP was detected using ATAC-seq and RNA-seq in iCCA specimens. Increased expression of PLK1 and MISP correlated with higher risks of tumor number, N stage, and lymphatic invasion in late-stage iCCA patients and with poor prognosis. Knockdown of PLK1 or MISP and treatment with a PLK1 inhibitor decreased lymphatic invasion and cell migration in vitro. Regarding the correlation between increased PLK1 and MISP expression and lymphatic invasion/poor patient survival, PLK1 or MISP knockdown increased E-cadherin dimerization, and dimerization was regulated by the ROCK inhibitor Y27632 and the MLCK inhibitor ML-7. Finally, we also confirmed the known role of PLK1 and MISP in cell proliferation in vitro and in vivo.

Vascular invasion, lymphatic invasion, and perineural invasion were significantly associated with worse OS in iCCA patients. Lymphatic invasion and N stage are considered poor prognostic factors for iCCA [52]. The expression of vascular endothelial growth factor-C (VEGF-C) in iCCA cells has been reported to be positively correlated with lymphatic invasion and tumor progression [53]. In this study, we demonstrated the crucial role of PLK1 and MISP in lymphatic invasion in iCCA. The major lymphatic invasion/metastatic processes are detachment from the primary tumor, penetration of lymphatic endothelial cells, and survival and growth in the lymph nodes. We found that PLK1 and MISP were involved in lymphatic invasion. This may be a vital issue for further study of iCCA aggressiveness.

PLK1 promoted EMT by controlling EMT-related proteins. In our study, PLK1 did not consistently affect EMT-related proteins in the two iCCA cell lines. PLK1 downregulated E-cadherin-mediated AJs through MLC phosphorylation in iCCA. Myosin-mediated contractility has been demonstrated to be involved in AJs [39, 41]. However, a few reports have shown the relationship between PLK1 and myosin activation. PLK1 can bind to some regulators of myosin activation, including myosin phosphatase-targeting subunit 1 (MYPT1) [54], ROCK2 [38], and supervillin [55]. These proteins may be involved in the PLK1-mediated myosin activation in iCCA.

Regarding the role of MISP in cell migration and AJs, MISP is an actin-binding protein and has been shown to localize and regulate focal adhesions via the interaction of focal adhesion kinase (Fig. 5E) [29]. For the regulation of AJs, dimerization of E-cadherin is modulated by the inside-out regulation of several proteins and factors, such as vinculin, cytoskeleton organization, and cytoskeletal tension [56]. As MISP is an actin-associated protein localized at AJs (Fig. 5E), MISP may thus modulate cytoskeletal architectures to impair AJs in iCCA.

Taken together, the results revealed that the cell cycle regulator PLK1 and its substrate MISP promoted lymphatic invasion and tumor growth and repressed E-cadherin adherens junctions, demonstrating the crucial role of PLK1 and MISP in iCCA aggressiveness.

### Supplementary information

supplementary information

Table S2

## Data Availability

Correspondence and requests for materials should be addressed to CN-Y (yehchunnan@gmail.com).
