# Supplementary material for: PLK1 and its substrate MISP facilitate intrahepatic cholangiocarcinoma progression by promoting lymphatic invasion and impairing E-cadherin adherens junctions
Source: Cancer Gene Ther. 2023 Dec 6;31(2):322–33. doi: 10.1038/s41417-023-00705-z (PMC10874889; doi:10.1038/s41417-023-00705-z)
Supplement: Supplementary file 1 — supplementary information [file 41417_2023_705_MOESM1_ESM.pdf]

Supplementary Figures

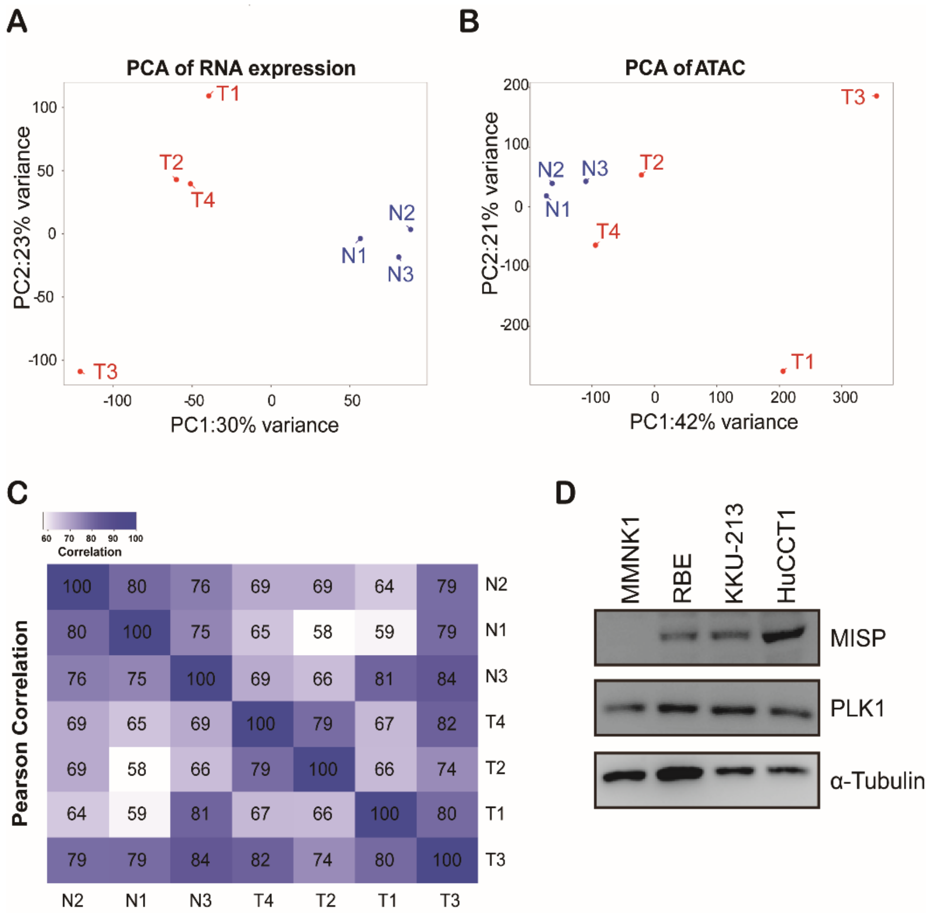

Supplementary Fig. 1. The association of PLK1 and MISP in HuCCT1 cells.

A. Comparison of the groups of normal (blue) and tumor (red) samples with RNA-seq datasets by principal component analysis. The results showed that the normal and tumor tissue groups could be separately clustered.

B. Comparison of the groups of normal (blue) and tumor (red) samples with ATAC-seq datasets by principal component analysis. The results showed that the normal and tumor tissue groups could be separately clustered.

C. The Pearson correlation plot of ATAC-seq shows the correlation between each sample.

D. Western blots showing the levels of MISP and PLK1 in MMNK, RBE, KKU-213, and HuCCT1 cells. α-Tubulin was the loading control.

A

	Hazard ratio	95 % C.I. lower	95 % C.I. upper
Gender: male vs. female	0.98	0.63	1.54
Age: ≦65 vs. >65	1.16	0.72	1.86
HBV infection: no vs. yes	1.95	1.13	3.39
HCV infection: no vs. yes	1.34	0.64	2.81
Bilirubin: >1.2 vs. ≦1.2	0.98	0.52	1.87
Albumin: ≦3.5 vs. >3.5	1.94	1.15	3.27
CEA: >5 vs. ≦5	1.33	0.83	2.11
Margin: positive vs. negative	2.30	1.41	3.75
Tumor no.: multiple vs. single	1.64	0.93	2.90
Gross type			
MF+PI vs. MF	2.19	1.27	3.78
PI vs. MF	2.01	1.01	3.99
Tumor size: >5cm vs. ≦5cm	1.40	0.88	2.21
T stage			
T2 vs. T1	2.10	1.12	3.93
T3/4 vs. T1	1.70	1.03	2.79
N stage: N1 vs. N0	2.33	1.45	3.74
Histology: undifferentiated vs. differentiated	1.27	0.81	1.99
Vascular invasion: yes vs. no	1.87	1.05	3.33
Lymphatic invasion: yes vs. no	1.48	0.87	2.52
Perineural invasion: yes vs. no	1.84	1.15	2.94
MISP1 H-score: high vs. low	2.08	1.32	3.28
PLK1 H-score: high vs. low	2.18	1.38	3.45

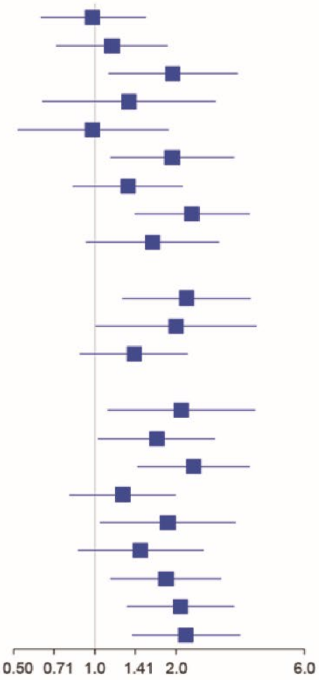

B

	Hazard ratio	95 % C.I. lower	95 % C.I. upper
HBV infection: no vs. yes	2.53	1.32	4.86
Albumin: ≦3.5 vs. >3.5	1.55	0.85	2.83
Margin: positive vs. negative	2.08	1.14	3.79
Gross type			
MF+PI vs. MF	2.08	1.08	4.03
PI vs. MF	1.85	0.85	4.00
T stage			
T2 vs. T1	1.92	0.72	5.12
T3/4 vs. T1	1.44	0.82	2.52
N stage: N1 vs. N0	2.36	1.29	3.12
Vascular invasion: yes vs. no	1.22	0.48	3.12
Perineural invasion: yes vs. no	0.80	0.46	1.41
MISP1 H-score: high vs. low	1.21	0.57	2.57
PLK1 H-score: high vs. low	1.89	0.88	4.07

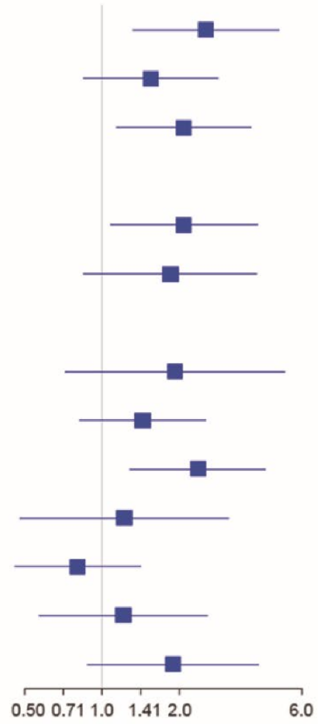

C

	Hazard ratio	95 % C.I. lower	95 % C.I. upper
Gender: male vs. female	1.06	0.68	1.65
Age: ≦65 vs. >65	1.37	0.86	2.19
HBV infection: no vs. yes	1.50	0.90	2.49
HCV infection: no vs. yes	1.73	0.83	3.61
Bilirubin: >1.2 vs. ≦1.2	1.18	0.63	2.18
Albumin: ≦3.5 vs. >3.5	1.62	0.97	2.72
CEA: >5 vs. ≦5	1.27	0.81	1.99
Margin: positive vs. negative	2.55	1.55	4.19
Tumor no.: multiple vs. single	1.41	0.80	2.49
Gross type			
MF+PI vs. MF	2.01	1.17	3.46
PI vs. MF	2.92	1.44	5.92
Tumor size: >5cm vs. ≦5cm	1.53	0.97	2.40
T stage			
T2 vs. T1	2.05	1.10	3.84
T3/4 vs. T1	1.85	1.13	3.01
N stage: N1 vs. N0	2.35	1.47	3.76
Histology: undifferentiated vs. differentiated	1.05	0.68	1.64
Vascular invasion: yes vs. no	1.76	0.99	3.12
Lymphatic invasion: yes vs. no	1.19	0.70	2.01
Perineural invasion: yes vs. no	1.86	1.17	2.97
MISP1 H-score: high vs. low	2.83	1.76	4.55
PLK1 H-score: high vs. low	3.39	2.08	5.53

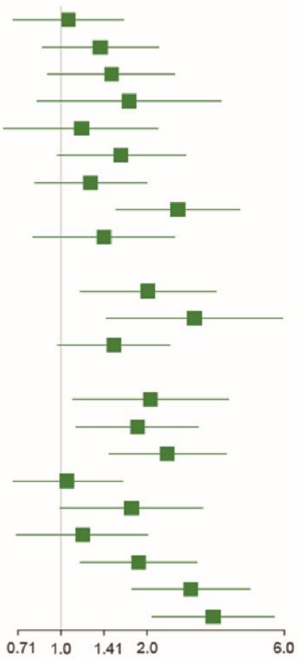

D

	Hazard ratio	95 % C.I. lower	95 % C.I. upper
Margin: positive vs. negative	1.78	0.99	3.21
Gross type			
MF+PI vs. MF	2.25	1.23	4.10
PI vs. MF	3.71	1.71	8.07
T stage			
T2 vs. T1	1.79	0.92	3.49
T3/4 vs. T1	1.86	1.05	3.30
N stage: N1 vs. N0	1.20	0.69	2.12
Perineural invasion: yes vs. no	1.16	0.68	1.99
MISP1 H-score: high vs. low	1.24	0.61	2.49
PLK1 H-score: high vs. low	3.10	1.48	6.53

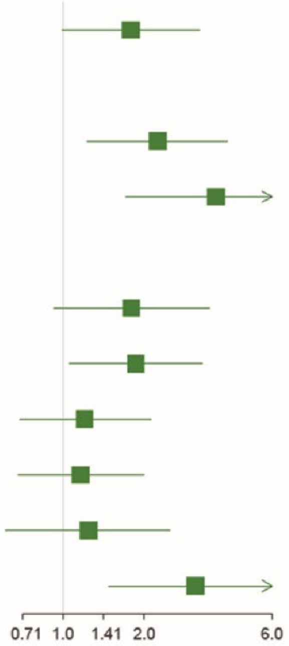

Supplementary Fig. 2. A Cox regression with multiple factors.

Forest plot based on univariate hazard ratios (**A** and **C**) or multivariate hazard ratios (**B** and **D**) from Cox regression for all subgroups after adjustment for centers. (**A** and **B**) Overall survival; (**C** and **D**) Disease-free survival. Squares represent hazard ratios. Bars represent 95% confidence intervals. The size of the square is proportional to the weights used in the analysis. Diamonds represent overall hazard ratios (center) with associated 95% confidence intervals.

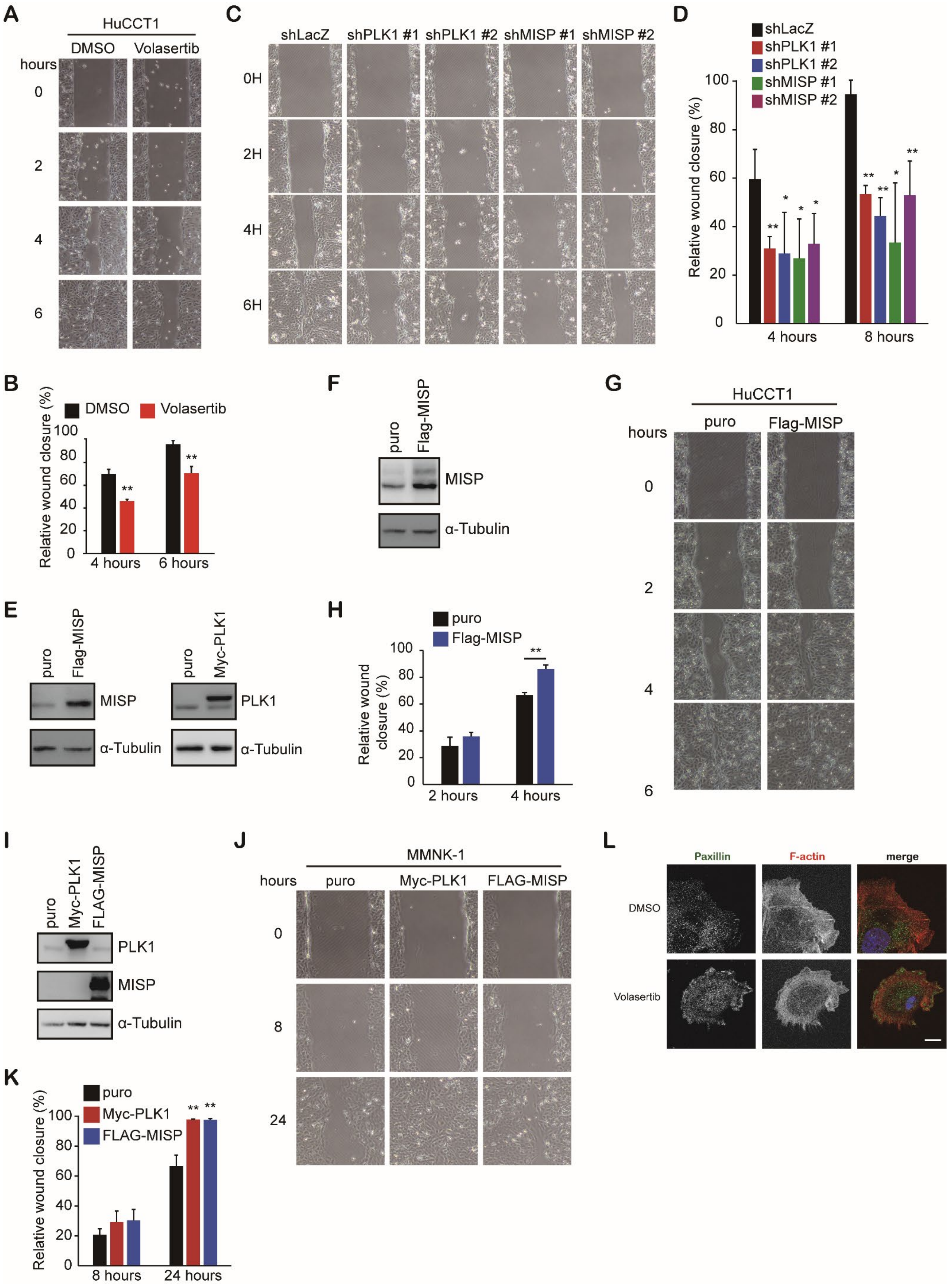
A
HuCCT1
DMSO
Volasertib
hours
0
2
4
6
B
Relative wound closure (%)
DMSO
Volasertib
4 hours
6 hours
C
shLacZ
shPLK1 #1
shPLK1 #2
shMISP #1
shMISP #2
0H
2H
4H
6H
D
shLacZ
shPLK1 #1
shPLK1 #2
shMISP #1
shMISP #2
Relative wound closure (%)
4 hours
8 hours
E
puro
Flag-MISP
MISP
α-Tubulin
puro
Myc-PLK1
PLK1
α-Tubulin
F
puro
Flag-MISP
MISP
α-Tubulin
G
HuCCT1
puro
Flag-MISP
hours
0
2
4
6
H
puro
Flag-MISP
Relative wound closure (%)
2 hours
4 hours
I
puro
Myc-PLK1
FLAG-MISP
PLK1
MISP
α-Tubulin
J
MMNK-1
hours
puro
Myc-PLK1
FLAG-MISP
0
8
24
K
puro
Myc-PLK1
FLAG-MISP
Relative wound closure (%)
8 hours
24 hours
L
Paxillin
F-actin
merge
DMSO
Volasertib

Supplementary Fig. 3. The manipulation of PLK1 or MISP expression regulated cell migration in iCCA cells.

A. Representative images from the wound-healing assay of HuCCT1 cells that received 1 μM volasertib or control DMSO treatment for 0, 2, 4, 6 or 8 hours.

B. The relative wound closure was calculated by analyzing the scratched area covered by the cells after four or six hours using ImageJ software. The values (means ± SDs) are from three independent experiments and are presented as a percentage relative to the baseline (0 hours). ** $P < 0.005$ by Student's *t* test.

C. Representative images from the wound healing assay of the HuCCT1-N4 subline that received shRNAs specific to PLK1 (shPLK1 #1 and #2), MISP (shMISP #1 and #2) or LacZ (shLacZ).

D. The relative wound closure was calculated by analyzing the scratched area covered by the cells after four or eight hours using ImageJ software. The values (means ± SDs) are from three independent experiments and are presented as a percentage relative to the baseline (0 hours). * $P < 0.05$, ** $P < 0.005$ by Student's *t* test.

E. Western blots showing the level of MISP in KKU-213 cells overexpressing vector (puro) or Flag-tagged wild-type MISP (Flag-MISP). α-Tubulin was the loading control. Right: Western blots showing the level of PLK1 in KKU-213 cells overexpressing vector (puro) or Myc-tagged PLK1 wild type (Myc-PLK1). α-Tubulin was the loading control.

F. Western blots showing the level of MISP in HuCCT1 cells overexpressing vector (puro) or Flag-tagged wild-type MISP (Flag-MISP). α-Tubulin was the loading control.

G. A wound-healing assay of vector (puro) or Flag-tagged wild-type MISP (Flag-MISP)-overexpressing HuCCT1 cells.

H. The relative wound closure was calculated by analyzing the scratched area covered by the cells after two or four hours using ImageJ software. The values (means ± SDs) are from three independent experiments and are presented as the fold change relative to the baseline (0 hours). ** $P < 0.005$ by Student's *t* test.

I. Western blots showing the level of MISP in MMNK-1 cells overexpressing vector (puro) or Flag-tagged wild-type MISP (Flag-MISP). α-Tubulin was the loading control.

J. A wound-healing assay of vector (puro) or Flag-tagged wild-type MISP (Flag-MISP)-overexpressing MMNK-1 cells.

K. The relative wound closure was calculated by analyzing the scratched area covered by the cells after two or four hours using ImageJ software. The values (means ± SDs) are from three independent experiments and are presented as the fold change relative to the baseline (0 hours). ** $P < 0.005$ by Student's *t* test.

L. HuCCT1 cells receiving 1 μM volasertib or control DMSO treatment for 6 hours were fixed and stained with paxillin (green), F-actin (red) and nuclear stain (blue). Scale bar = 10 μm.

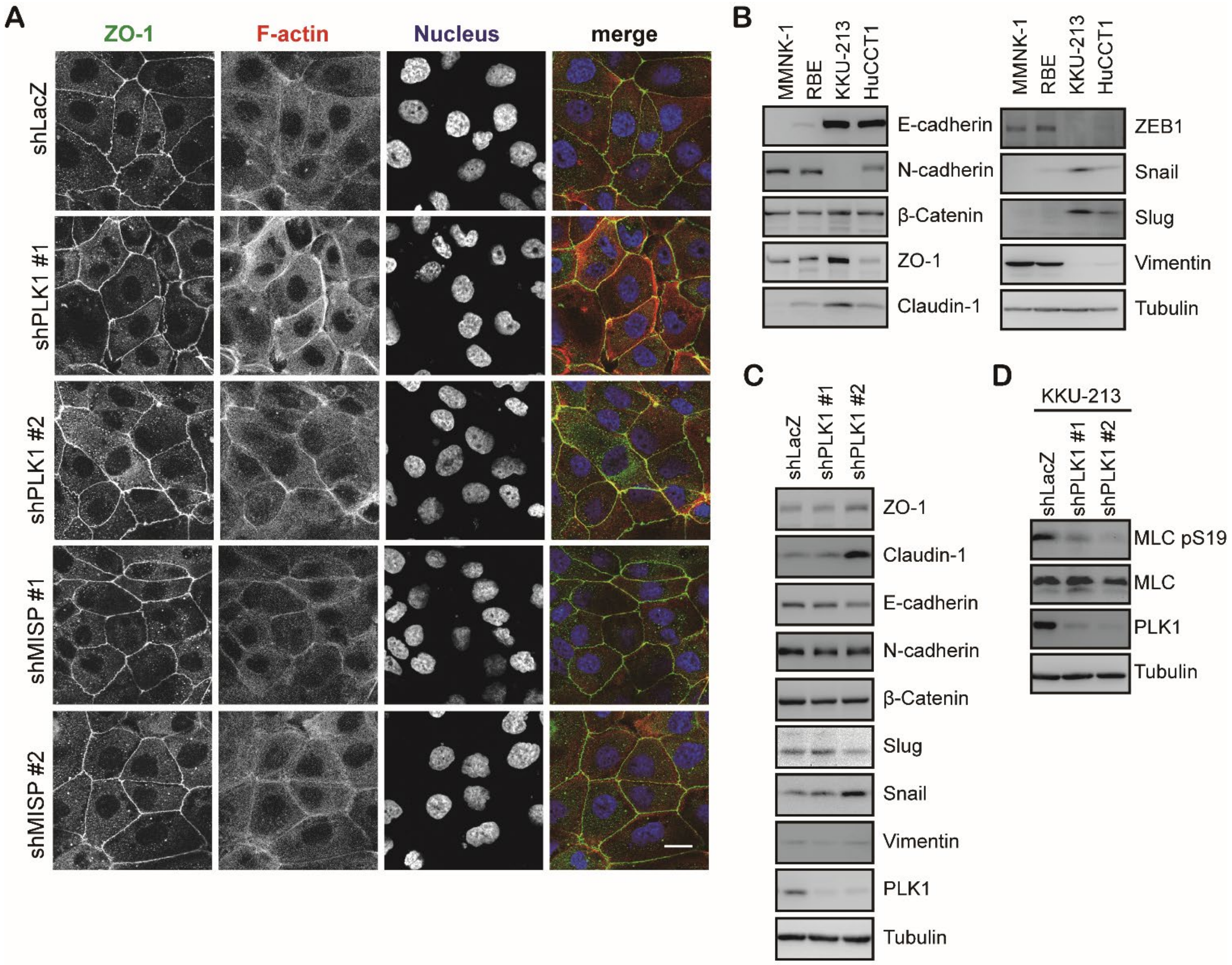

Supplementary Fig. 4. The effect of PLK1 and MISP on the localization of ZO-1 and the expression of EMT-related proteins.

A. HuCCT cells that received shRNAs specific to PLK1 (shPLK1 #1 and #2), MISP (shMISP #1 and #2) or LacZ (shLacZ) were grown to confluence and stained with ZO-1 (green), F-actin (red) and nuclear stain (blue). Scale bar = 10 μm.

B. Western blots showing the levels of the indicated proteins in MMNK, RBE, KKU-213 and HuCCT1 cells. α-Tubulin was the loading control.

C. Western blots showing the levels of the indicated proteins in HuCCT1 cells that received shRNAs specific to PLK1 (shPLK1 #1 and #2) or LacZ (shLacZ). α-Tubulin was the loading control.

D. Western blots showing the levels of the indicated proteins in KKU-213 cells that received shRNAs specific to PLK1 (shPLK1 #1 and #2) or LacZ (shLacZ). α-Tubulin was the loading control.

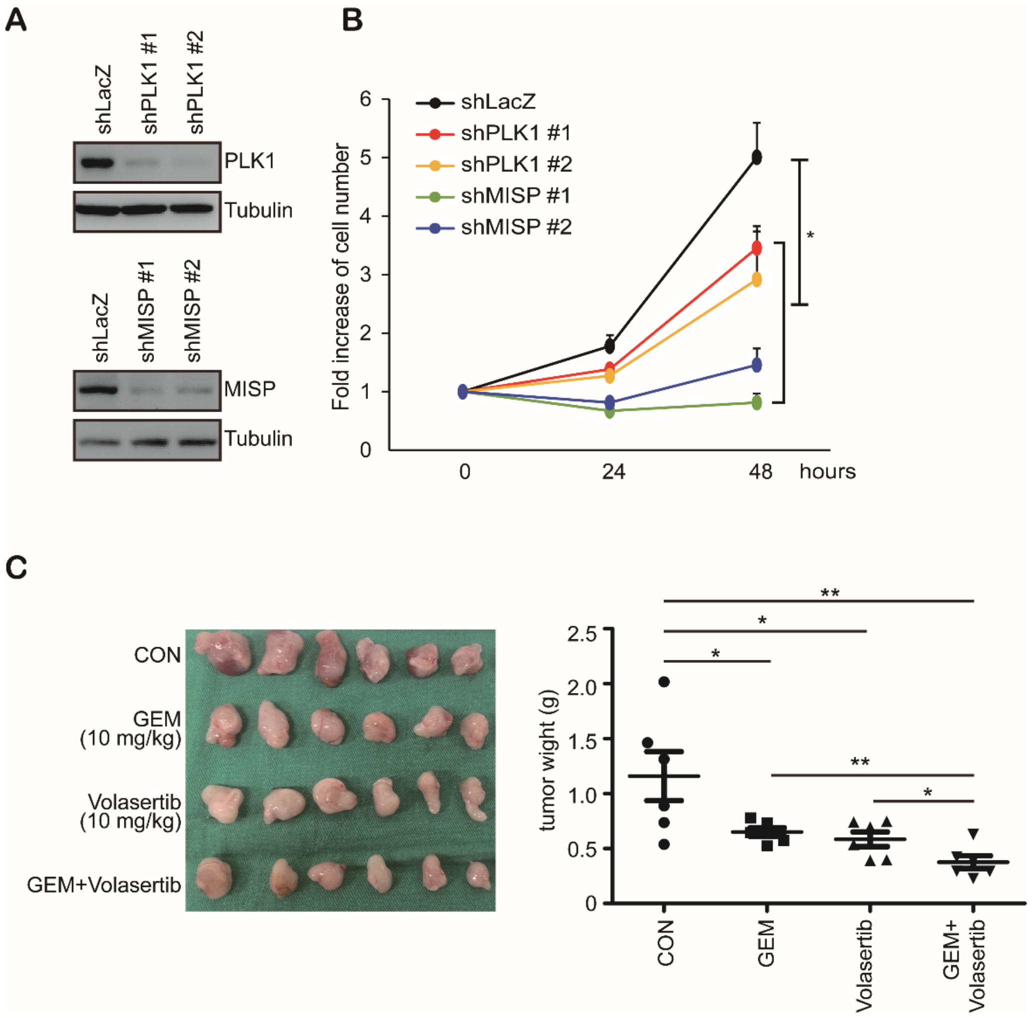

Supplementary Fig. 5. Knockdown of PLK1 or MISP suppressed cell proliferation in KKU-213 cells.

A. Western blots showing the levels of PLK1 and MISP in KKU-213 cells that received shRNAs specific to PLK1 (shPLK1 #1 and #2), MISP (shMISP #1 and #2) or LacZ (shLacZ).

B. Cell proliferation assay. A total of 5×10^3 cells were seeded in a 96-well plate for 0, 24 or 48 hours. Cell viability was quantified by the CCK-8 assay. The values (means ± SDs) are from three independent experiments and are presented as the fold change relative to the baseline (0 h). * $P < 0.05$ by Student's *t* test.

C. The xenograft animal model was treated with or without gemcitabine (GEM) and volasertib. HuCCT1 cells were injected into the subcutaneous tissue of BALB/c nude mice. The mice were given 10 mg/kg GEM weekly by intraperitoneal injection and 10 mg/kg volasertib by intravenous injection twice per week for 3 weeks. After 3 weeks, the mice were sacrificed, and the tumor images (left) and tumor volumes (right) are shown. n=6 for each group. The values are presented as the means ± SEMs. *$P < 0.05$, **$P < 0.005$ by Student's *t* test.

Supplementary Materials and Methods

ATAC-sequencing

Frozen tissue was homogenized using a Dounce homogenizer. After releasing the nuclei by douncing, the nuclear band was extracted at the interface between 29% and 35% iodixanol solutions. The tagmentation reaction was performed at 37°C using a ThermoMixer (1000 rpm for 30 minutes). Tagmented products were collapsed using a Zymo DNA Clean and Concentrator-5 kit. The library was constructed using NEBNext® High-Fidelity 2X PCR Master Mix (New England Biolabs, Ipswich, MA, USA), and PCR amplification was performed according to the previous methodology [1]. The PCR product was purified using Ampure XP beads (Beckman Coulter Inc., Brea, CA, USA). The constructed library was subjected to quality control by Tapstation and then sequenced with Next-Seq 550 by 75-bp paired-end sequencing.

RNA sequencing

Standard protocols were followed for RNA extraction. For RNA sequencing, we selected four normal tissues and four tumor tissues from patients with CCA. RNA sequencing libraries were prepared using a KAPA RNA HyperPrep Kit with RiboErase and were sequenced with Illumina Nextseq 550 to obtain 150-bp paired-end reads. The datasets were deposited in the Gene Expression Omnibus (GEO) database under accession number GSE244331. For quantitative RT–PCR, RT–qPCR was performed as previously described [2], and the primers used in this study are shown in Supplementary Table 3.

Sequencing data analysis

For ATAC-seq, reads were trimmed using Trim Galore. Data were aligned using Bowtie2 against the hg38 reference genome[3]. After alignment, files were filtered according to mapping quality, and PCR duplicates were removed. Chromatin-accessible region peaks were called using MACS2[4] and filtered to a q value < 0.01. To perform differential chromatin accessible region analysis, we applied diffbind, R package, to find the consensus accessible regions across samples and then counted and normalized the ATAC-seq reads of each region. plotPCA was used to sort the principal components according to the amount of data variability. The pairwise Spearman correlation between any pair of ATAC-seq samples was calculated based on read counts/signals on merged ATAC-seq peaks from all samples. Heatmaps and profile plots were generated using DeepTools. A visualization of the genome-wide read coverage was generated by converting raw BAM files to bigWig files using IGV tools. For RNA-seq, the reads were trimmed using Trim Galore. The data were aligned using HISAT2 against the hg38 reference genome. After alignment, the files were filtered according to mapping quality higher than 10. Counts of each gene were generated by htseq-count, and differential analysis was performed by edgeR, R package.

Recombinant plasmids and reagents

The pCDH-puro-Flag-MISP and pCDH-puro-Myc-PLK1 expression vectors were generated by inserting full-length wild-type MISP (NM_173481.4) or full-length wild-type PLK1 (NM_005030.3) [5] into the pCDH-CMV-MCS-EF1α-Puro vector (CD510B-1, System Biosciences, LLC, Palo Alto, CA). Volasertib was purchased from AbMole BioScience (M1678, Houston, USA). Gemcitabine was purchased from TTY Biopharm Company Limited (Taiwan). Y27632 and ML-7 were purchased from Selleck Chemicals (Houston, USA).

Virus production and infection

Virus production and infection were performed as previously described [2]. In brief, pCMV-ΔR8.91, pMD.G, pLKO.1-shRNA clones (Supplementary Table 4; the National RNAi Core Facility, Academia Sinica, Taiwan), pCDH-puro-Flag-MISP or pCDH-puro-Myc-PLK1 were cotransfected into HEK293T cells using jetPEI (Polyplus Transfection, New York, NY, USA). The lentivirus particles were collected after 48 and 72 hours. The cells were incubated in virus-containing media with 8 μg/ml polybrene (Sigma–Aldrich, St. Louis, MO) for 24 hours and then selected by puromycin for another three days.

Immunoprecipitation and immunoblotting

For immunoprecipitation, the cells were lysed in 1% Nonidet P-40 lysis buffer (150 mM NaCl, 1% NP-40, 50 mM Tris, pH 7.4) plus protease inhibitors (Roche, Mannheim, Germany) and incubated on ice for at least 30 minutes. Immunoblotting was performed as previously described [2]. In brief, the cells were lysed in RIPA buffer (150 mM NaCl, 1.0% NP-40, 0.5% sodium deoxycholate, 0.1% SDS, 50 mM Tris, pH 7.5) plus protease inhibitors, incubated on ice for at least 30 minutes and centrifuged at 13,000 rpm for 10 minutes at 4°C. The proteins were separated by SDS–PAGE gels and transferred onto PVDF membranes (Millipore, Billerica, MA). The membranes were blocked, incubated with the primary antibodies (Supplementary Table 5), and incubated with secondary antibodies. The results were observed and analyzed using UVP ChemStudio PLUS Touch (Analytik Jena AG, Jena, Germany).

Immunofluorescence staining and analysis

Immunofluorescence staining was performed as previously described [2]. In brief, the cells were fixed with 4% paraformaldehyde, permeabilized with 0.5% Triton X-100 for 30 min at room temperature and blocked with 4% FBS in PBS for 2 hours. The cells were incubated with the primary antibodies (Supplementary Table 4) in 4% FBS at 4°C overnight, secondary antibodies at 4°C overnight (Thermo Fisher Scientific, Inc., Waltham, MA, United States), and phalloidin (Thermo Fisher Scientific, Inc., Waltham, MA, United States) for 30 min at room temperature. Coverslips were applied for mounting in mounting medium with DAPI-Aqueous, Fluoroshield (Abcam Plc., Cambridge, United Kingdom), and the cells were viewed using a laser-scanning confocal microscope image system (Leica TCS SP8X, Leica Microsystems, Wetzlar, Germany). The areas of focal

adhesions = $\pi\times$ semimajor axis $\times$ semiminor axis. The relative fluorescence intensity of E-cadherin (Fig. 5A, B, I, J) was analyzed using Leica Application Suite X (LAS X) software.

Proliferation

For the proliferation assay, the cells (5×10^3) were seeded in 96-well plates for 0, 24, 48, and 72 hours. Cell viability was quantified by a CCK-8 assay (Dojindo Molecular Technologies, Inc., Kumamoto, Japan) according to the manufacturer's instructions.

Translymphatic endothelial migration assay

A translymphatic endothelial migration assay was performed as previously described [6]. In brief, 4×10^5 HDLECs were added to the upper 24-well Transwell chamber with 8-μm pores for 24 hours. The next day, 5×10^4 or 2×10^4 CCA cells were added to the upper chamber in 250 μl of serum-free medium, and the lower chamber was loaded with 750 μl of medium with 10% serum. After 24 hours, the migrated cells were fixed with 100% methanol, stained with 10% Giemsa stain, and counted.

Wound healing assay

The cells (5×10^4) were seeded in one well of culture inserts (80209, ibidi, Wisconsin, USA). After 24 hours, the culture inserts were removed, and images were captured at 0, 2, 4, 6, and 8 hours. The wound closure areas were measured with ImageJ, and the results are presented as a percentage relative to the area at 0 hours.

Supplementary references

1. Corces MR, Trevino AE, Hamilton EG, Greenside PG, Sinnott-Armstrong NA, Vesuna S, et al. An improved ATAC-seq protocol reduces background and enables interrogation of frozen tissues. Nat Methods. 2017; 14: 959-62.
2. Pan YR, Wu CE, Yeh CN. ATM Inhibitor Suppresses Gemcitabine-Resistant BTC Growth in a Polymerase theta Deficiency-Dependent Manner. Biomolecules. 2020; 10.
3. Langmead B, Salzberg SL. Fast gapped-read alignment with Bowtie 2. Nat Methods. 2012; 9: 357-9.
4. Zhang Y, Liu T, Meyer CA, Eeckhoute J, Johnson DS, Bernstein BE, et al. Model-based analysis of ChIP-Seq (MACS). Genome Biol. 2008; 9: R137.
5. Golsteyn RM, Schultz SJ, Bartek J, Ziemiecki A, Ried T, Nigg EA. Cell cycle analysis and chromosomal localization of human Plk1, a putative homologue of the mitotic kinases Drosophila polo and Saccharomyces cerevisiae Cdc5. J Cell Sci. 1994; 107 (Pt 6): 1509-17.
6. Naidoo K. A simple human co-culture model of lymphatic invasion. J Clin Pathol. 2017; 70: 266-9.

Supplementary Table 1. Ten fresh tissues used in this study

Patient ID	Tissue Sample	Tissue ID	Sex	Pathology report	ATAT-seq	RNA-seq	RT–qPCR
Patient 1	Tumor	T1	Female	iCCA	●	●	●
Patient 1	Adjacent normal liver tissue	N1	Female	iCCA	●	●	●
Patient 2	Tumor	T2	Female	iCCA	●	●	●
Patient 2	Adjacent normal liver tissue	N2	Female	iCCA	●	●	●
Patient 3	Tumor	T3	Male	iCCA	●	●	●
Patient 3	Adjacent normal liver tissue	N3	Male	iCCA	●	●	●
Patient 4	Tumor	T4	Male	iCCA	●	●	●
Patient 4	Adjacent normal liver tissue	N4	Male	iCCA			●
Patient 5	Tumor	T5	Female	iCCA			●
Patient 5	Adjacent normal liver tissue	N5	Female	iCCA			●

iCCA: Intrahepatic cholangiocarcinoma

Supplementary Table 3. Information on PCR primers

Gene		Sequence (5'-3')
GAPDH	F	GTCTCCTCTGACTTCAACAGCG
	R	ACCACCCTGTTGCTGTAGCCAA
PLK1	F	ACTTCGTGTTCGTGGTGTTGG
	R	GCTTGAGGTCTCGATGAATAAC
MISP	F	CACCTACACTCAAACGTGGCGT
	R	CCTCTGAGTTGATACCGTCCGA

Supplementary Table 4. Information on shRNA sequences

Gene name	shRNA target sequence
LacZ	CGCGATCGTAATCACCCGAGT
PLK1 #1	GTTCTTTACTTCTGGCTATAT
PLK1 #2	GCTCATCTTGTGCCCACTGAT
MISP #1	GACACCAGCTACACATACCAT
MISP #2	GCGCTGGGAATCCCGCATCTA

Supplementary Table 5. Information on the antibodies used in this study

Protein	Application (dilution)	Catalog No.	Origin	Company
α-Tubulin	WB (1:5000)	T6793	mouse mAb	Sigma–Aldrich (St. Louis, MO)
beta-Catenin	WB (1:2000)	#8480	rabbit mAb	Cell Signaling Technology, Inc. (Danvers, MA)
Claudin-1	WB (1:1000)	#13255	rabbit mAb	Cell Signaling Technology, Inc. (Danvers, MA)
MISP	IHC (1:1500)	ab254919	rabbit pAb	Abcam plc. (Cambridge, UK)
MISP	WB (1:2000); IF (1:50)	26338-1-AP	rabbit pAb	Proteintech Group, Inc. (Rosemont, USA)
MLC pS19	WB (1:1000)	#3671	rabbit pAb	Cell Signaling Technology, Inc. (Danvers, MA)
MLC pS19	IHC (1:400)	600-401-416	rabbit pAb	Thermo Fisher Scientific Inc. (Waltham, MA)
MLC	WB (1:1000)	#8505	rabbit mAb	Cell Signaling Technology, Inc. (Danvers, MA)
E-Cadherin (24E10)	WB (1:2000); IF (1:100)	#3195	rabbit mAb	Cell Signaling Technology, Inc. (Danvers, MA)
E-Cadherin (ECCD-2)	IF (1:200)	#13-1900	rat mAb	Thermo Fisher Scientific Inc. (Waltham, MA)
FAK pY397	WB (1:1000)	#3283	rabbit pAb	Cell Signaling Technology, Inc. (Danvers, MA)
FAK	WB (1:2000)	#3285	rabbit pAb	Cell Signaling Technology, Inc. (Danvers, MA)
N-Cadherin	WB (1:2000)	#5741	rabbit mAb	Cell Signaling Technology, Inc. (Danvers, MA)
Histone H3 pS10	IF (1:800)	#9701	rabbit pAb	Cell Signaling Technology, Inc. (Danvers, MA)
PLK1	WB (1:2000); IHC (1:100); IF (1:50)	#37-7000	mouse mAb	Thermo Fisher Scientific Inc. (Waltham, MA)
Paxillin pY118	WB (1:1000)	#2541	rabbit pAb	Cell Signaling Technology, Inc. (Danvers, MA)
Paxillin	WB (1:2000); IF (1:200)	610051	mouse mAb	BD Transduction Laboratories™ (Franklin Lakes, NJ)
Snail	WB (1:1000)	#3879	rabbit mAb	Cell Signaling Technology, Inc. (Danvers, MA)
Slug	WB (1:1000)	#9585	rabbit mAb	Cell Signaling Technology, Inc. (Danvers, MA)

Vimentin	WB (1:1000)	#5741	rabbit mAb	Cell Signaling Technology, Inc. (Danvers, MA)
ZEB1	WB (1:1000)	#3396	rabbit mAb	Cell Signaling Technology, Inc. (Danvers, MA)
ZO-1	WB (1:1000)	#8139	rabbit mAb	Cell Signaling Technology, Inc. (Danvers, MA)
ZO-1	IF (1:100)	#33-9100	mouse mAb	Thermo Fisher Scientific Inc. (Waltham, MA)

Abbreviations: WB, wWestern blot; IF, immunofluorescence; IHC, immunohistochemistry; mAb: monoclonal antibody; pAb, polyclonal antibody
